# Quantitative in vivo assessment of radiation injury of the liver using Gd-EOB-DTPA enhanced MRI: tolerance dose of small liver volumes

**DOI:** 10.1186/1748-717X-6-40

**Published:** 2011-04-17

**Authors:** Max Seidensticker, Ricarda Seidensticker, Konrad Mohnike, Christian Wybranski, Thomas Kalinski, Sebastian Luess, Maciej Pech, Peter Wust, Jens Ricke

**Affiliations:** 1 Klinik für Radiologie und Nuklearmedizin, Universitätsklinikum Magdeburg, Otto-von-Guericke-Universität Magdeburg, Germany; 2 Institut für Pathologie, Universitätsklinikum Magdeburg, Otto-von-Guericke-Universität Magdeburg, Germany; 3 Klinik für Strahlentherapie, Charité Universitätsmedizin Berlin, Campus Virchow Klinikum, Germany

## Abstract

**Backround:**

Hepatic radiation toxicity restricts irradiation of liver malignancies. Better knowledge of hepatic tolerance dose is favourable to gain higher safety and to optimize radiation regimes in radiotherapy of the liver. In this study we sought to determine the hepatic tolerance dose to small volume single fraction high dose rate irradiation.

**Materials and methods:**

23 liver metastases were treated by CT-guided interstitial brachytherapy. MRI was performed 3 days, 6, 12 and 24 weeks after therapy. MR-sequences were conducted with T1-w GRE enhanced by hepatocyte-targeted Gd-EOB-DTPA. All MRI data sets were merged with 3D-dosimetry data. The reviewer indicated the border of hypointensity on T1-w images (loss of hepatocyte function) or hyperintensity on T2-w images (edema). Based on the volume data, a dose-volume-histogram was calculated. We estimated the threshold dose for edema or function loss as the D_90_, i.e. the dose achieved in at least 90% of the pseudolesion volume.

**Results:**

At six weeks post brachytherapy, the hepatocyte function loss reached its maximum extending to the former 9.4Gy isosurface in median (i.e., ≥9.4Gy dose exposure led to hepatocyte dysfunction). After 12 and 24 weeks, the dysfunctional volume had decreased significantly to a median of 11.4Gy and 14Gy isosurface, respectively, as a result of repair mechanisms. Development of edema was maximal at six weeks post brachytherapy (9.2Gy isosurface in median), and regeneration led to a decrease of the isosurface to a median of 11.3Gy between 6 and 12 weeks. The dose exposure leading to hepatocyte dysfunction was not significantly different from the dose provoking edema.

**Conclusion:**

Hepatic injury peaked 6 weeks after small volume irradiation. Ongoing repair was observed up to 6 months. Individual dose sensitivity may differ as demonstrated by a relatively high standard deviation of threshold values in our own as well as all other published data.

## Backround

Irradiation of liver malignancies has evolved as an effective treatment alternative to liver surgery in selected patients. Both external radiotherapy as well as image guided brachytherapy have been described in the literature with promising results [1-4]. One of the few limiting factors is the tolerance dose of the surrounding liver parenchyma.

Literature of quantitative in vivo data of the hepatic tolerance to irradiation is limited [5-8]. However, such knowledge is essential for the treatment strategy in patients with multiple or large tumors or in situations with a small parenchymal reserve after liver resection.

Previous studies on the tolerance dose of the liver are mainly based on fractionated large volume liver irradiation with the clinical endpoint of radiation induced liver disease (RILD). This status may occur if more than 30 - 55 Gy are applied, depending on the irradiated liver volume [6,7,9-14]. These doses lead to a decline of the total organ function causing clinical symptoms. However, this does not necessarily reflect the inherent radiosensitivity of the liver and even less the intrinsic radiosensitivity of hepatocytes or the liver functioning units.

Computed tomography (CT)-guided brachytherapy of liver malignancies utilizes CT fluoroscopy for catheter positioning and three dimensional (3D) CT-data sets for dose planning. During follow up after irradiation, magnetic resonance imaging (MRI) may be used as a sensitive method to detect edema as well as liver function loss by employing hepatocyte-directed contrast agents. By applying image fusion of follow up MRI with the treatment planning CT, the isodoses calculated for interstitial irradiation can be displayed in the MRI scans, indicating the exact dose distribution at any given time point during follow up. In a precursor study, we utilized this approach along with the hepatocyte directed contrast agent Gadobenate dimeglumine (Gd-BOPTA). We determined a median threshold dose of 9.9 Gy after six weeks as the minimal tolerance dose of small volume liver parenchyma to single fraction high dose rate (HDR) brachytherapy over time [8].

However, Gd-BOPTA displays only a small biliary excretion of only 6%. In contrast, Gadolinium ethoxybenzyl diethylenetriamine-pentaacetate (Gd-EOB-DTPA), a second generation hepatocyte directed contrast agent, has shown vast improvements over Gd-BOPTA in liver contrast through biliary excretion rates of >50% [15-18]. By employing Gd-EOB-DTPA in the study described herein we sought to determine the hepatic tolerance dose to small volume single fraction high dose irradiation as primary endpoint. As secondary endpoints we searched for factors of influence on the threshold dose (history of chemotherapy, irradiated volume etc.) and we intended to gain a more accurate assessment of dose thresholds specifically in light of the relatively high standard deviation in the precursor study employing Gd-BOPTA. As with Gd-BOPTA, surrogate for local liver function was a diminished uptake of the contrast agent in liver parenchyma, and image fusion with dosimetry data determined the respective threshold doses.

## Material and methods

### Patient identification

Twenty-three patients were included in this study. All patients were scheduled to receive a CT guided HDR single fraction brachytherapy of one liver malignancy each. Follow-up MRI employed Gd-EOB-DTPA. The study was approved by the institutional ethics committee.

The patient population comprised of 12 men and 11 women. The mean age was 66 years (30-84 years). All patients demonstrated a Karnofsky score greater than 80%. All liver tumors were metastastic, and liver cirrhosis was exclusion criteria. The primary tumors were: 10 colorectal, 8 breast, 3 renal cell, 1 gastric and 1 non small cell lung cancer (Table 1).

**Table 1:** Patient identification and previous cancer therapies

Patient	Age -yr	Primary Tumor site	Treatment date (months after first diagnosis)	Liver Volume -ccm	CTV -ccm	With ≥10 Gy irradiated Liver Volume -ccm	Chemotherapy prior to brachytherapy	Chemotherapy during follow-up	Liver resection or local treatment prior to brachytherapy
1	84	Colon	79	1063	66.7	249.5	n/a	none	Right hemihepatectomy, RFA
2	69	Gastric	16	1720	340.4	689	CAP+DOC, CAP	none	none
3	66	Lung	10	2135	30.6	205	none	GEM	RFA
4	66	Colon	13	1296	3.64	19	FOLFOX	none	none
5	66	Breast	83	1206	2.7	79.5	TAM, END+EPI+5FU/FA, EXE	EXE	none
6	63	Breast	18	1301	41.5	277.7	VP 16+JM8, DOC	GEM, DOC+CAP	none
7	72	Colon	30	1499	23.1	141	5FU/FA, FOLFOX	none	Wedge resection S4
8	30	Breast	12	1334	9.2	90.6	DOC+EPI, TAM+LEU, VIN+ Anti-Her-2/neu, 5FU/FA	CAP	none
9	61	Breast	n/a	1406	15.1	91.3	none	none	Wedge resection S4
10	70	Colon	14	2672	20.5	181	5FU/FA	none	none
11	58	Colon	49	1531	36.4	236	5FU/FA, FOLFIRI, FOLFOX	none	none
12	69	Colon	43	1610	100.7	381.6	FOLFIRI, FOLFOX, 5FU/FA, Anti-EGFR +CPT11	Anti-EGFR+CPT11	none
13	61	Colon	n/a	1350	123.6	327.6	FOLFOX+Anti-VEGF, 5FU/FA,	FOLFOX	Right hemihepatectomy, RFA
14	72	Renal	n/a	1170	1.7	49.4	none	none	Wedge resection, RFA
15	55	Colon	56	1484	58.5	370	FOLFIRI, FOLFOX	none	Right hemihepatectomy
16	62	Colon	20	1247	4.9	104.5	FOLFOX	none	none
17	56	Renal	6	822	30.5	137.7	none	SOR	none
18	55	Colon	22	1170	7.3	145	CAP+L-OHP, CAP+L-OHP+ Anti-VEGF	none	Right hemihepatectomy
19	69	Breast	34	1073	10.1	60.1	EPI+DOC, Anti-Her-2/neu +CAP+VIN, SDX 105, DOC	none	none
20	53	Breast	125	1054	0.8	22.2	VP 16+CAR, DOC+ADR, TAM, EXE, LET, 5FU/FA+CTX+EPI, FUL, GEM	none	none
21	52	Breast	16	1650	7.1	102	VP 16+JM8, LET	CAP	none
22	76	Renal	156	930	2.9	14.7	none	none	Wedge resection, RFA
23	77	Breast	80	1503	28.9	100.7	CAP	none	none

Abbreviations: Bendamustine (SDX 105), Bevacizumab (Anti-VEGF), Capecitabine (CAP), Carboplatin (JM8), Cetuximab (Anti-EGFR), Cyclophosphamide (CTX), Docetaxel (DOC), Doxorubicin (ADR), Endoxane (END), Epirubicin (EPI), Etoposide (VP 16), Exemestan (EXE), 5-Fluorouracil (5FU), Folic acid (FA), 5-Fluorouracil/Folic acid +Irinotecan (FOLFIRI), 5-Fluorouracil/Folic acid +Oxaliplatin (FOLFOX), Fulvestrant (FUL), Gemcitabine (GEM), Irinotecan (CPT 11), Letrozole (LET), Leuprorelin (LEU), Oxaliplatin (L-OHP), Sorafenib (SOR), Tamoxifen (TAM), Trastuzumab (Anti-Her-2/neu), Vinorelbine (VIN).

Combined applications are marked by +. Comma marks indicate sequential chemotherapeutic regimens.

Clinical Target Volume (CTV), Radiofrequency Ablation (RFA)

### Eligibility criteria

In addition to patients with clinical signs of liver cirrhosis we excluded patients who had previously undergone radiotherapy of the liver. To avoid confounding radiosensitizing effects or toxicities, systemic chemotherapy was paused for at least 14 days prior and post brachytherapy (Table 1).

### Interventional technique and follow up

The technique of CT-guided brachytherapy has been described in detail elsewhere [3,4]. In brief, placement of the brachytherapy applicators was performed under CT fluoroscopy. For treatment planning purposes, a spiral CT of the liver (slice thickness: 5 mm; increment: 5 mm) enhanced by intravenous administration of iodide contrast media (100 ml Ultravist 370, Bayer Schering Pharma, Berlin, Germany, flow: 1 ml/s; start delay: 80s) was acquired after positioning of the brachytherapy catheters in the tumor. A median of three catheters was used in our patients (range: 1 - 7 catheters).

**Figure 1:**
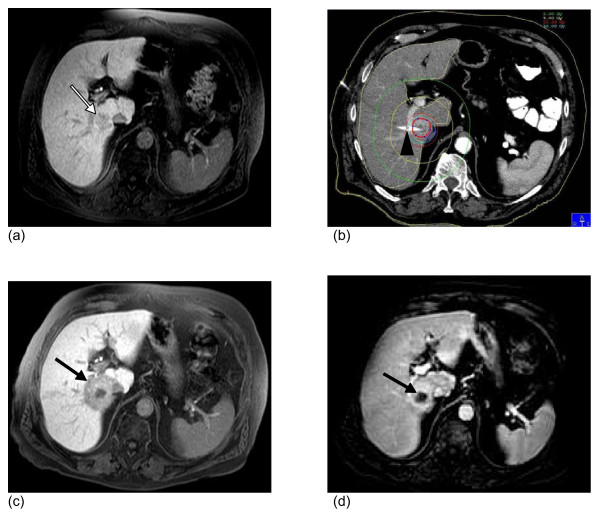
**Illustration of CT guided brachytherapy and post interventional hepatic dysfunction in MRI**. (a) Baseline MRI. T1-w GRE 20 minutes post i.v. application of Gd-EOB-DTPA. Colorectal liver metastasis in segment 6/7 (white arrow). (b) Contrast-enhanced computed tomography (CT) after CT-guided positioning of one brachytherapy catheter (truncated, black arrowhead) in the metastasis. The red line resembles the 15Gy isodose. (c) MRI 6 weeks after treatment. T1-w GRE 20 minutes post i.v. application of Gd-EOB-DTPA. Signal void around the tumor indicates hepatocyte dysfunction of liver parenchyma (black arrow). No evidence of tumor regrowth in (d): T1-w GRE dynamic scan 60s after application of the contrast dye shows shrinkage resulting from tumor necrosis after irradiation (black arrow).

The planning CT data set was digitally transferred to the treatment planning unit (BrachyVision^®^, Varian Medical Systems, Charlottesville, VA, USA). A radiologist defined the Clinical Target Volume (CTV) in the planning CT data set (Figure 1b). An one day prior to treatment obtained MRI of the liver was taken visually into account to avoid underestimation of the tumor size. To fulfill dosimetry planning in a timely manner, no registration of pre-treatment MRI with planning CT was performed due to patient safety and patient comfort. Based on literature and on own, yet unpublished data, the prescribed minimal dose inside the CTV was 15 to 20 Gy [4]. The true D100 applied was 14.3 to 21.2 (median 20Gy). The CTV ranged from 0.8 ml to 340.4 ml (median 20.5 ml), the volume (tumor plus liver parenchyma) which was exposed to more than 10 Gy ranged from 14.7 - 689 ml (median 137.7 ml) (Table 1).

The high dose rate afterloading system employed a ^192^Iridium source of 10Ci (Gammamed^®^, Varian medical systems, Charlottesville, VA, USA). The source diameter was < 1 mm. Dwell positions were located every 5 mm. Dwell times were corrected automatically according to the actual source strength. The median duration of irradiation was 1154 seconds (range: 250 to 3762 seconds).

The calibration factor used to compensate for the decay of the ^192^Iridium source ranged from 0.88 to 1.65 (median 1.36) relative to 10Ci.

Baseline MRI (Gyroscan NT^® ^1.5T, Philips, Best, The Netherlands) was obtained in all patients one day prior to therapy. During follow-up, MRI was performed at 3 days, 6, 12 and 24 weeks after treatment. The MRI protocol consisted of the following sequences: T2-weighted (T2-w) ultrafast spinecho (UTSE) (time to echo (TE)/time to repetition (TR) 90/2100 ms) with and without fat suppression, T1-weighted (T1-w) gradient echo (GRE) (TE/TR 5/30 ms, flip angle 30°) pre-contrast, 20s, 60s and 120s post intravenous administration of 0.025 mmol/kg bodyweight Gd-EOB-DTPA (Primovist^®^, Bayer Schering Pharma, Berlin, Germany), and 20 minutes post injection of intravenous Gd-EOB-DTPA. The slice thickness was 5 mm (T1-w) and 8 mm (T2-w) acquired in interleafed mode with no gap applied.

### Gd-EOB-DTPA

We used the diminished uptake of the hepatocyte specific contrast agent Gd-EOB-DTPA as a surrogate marker for the functioning state of the liver parenchyma. Gd-EOB-DTPA is a newly developed water soluble MR contrast agent containing a lipophilic moiety. Like other gadolinium contrast media, the contrast function is basically determined by the paramagnetic gadolinium ion leading to a high T1 relaxivity. Unlike common MRI contrast agents (e.g. Gd-DTPA), Gd-EOB-DTPA is distributed not only to the extracellular fluid space, but taken up by the organic anion transporting polypeptide (OATP) of the hepatocytes. It is excreted via the canalicular multispecific organic anion transporter (cMOAT/mrp2) following a linear, concentration dependent mechanism [19-22]. Animal studies have shown a biliary excretion rate of 63-80% and 32-34% in rats and simians, respectively. Biodistribution studies in humans reveal a dose independent biliary (41.6-51.2%) and renal (43.1-53.2%) elimination and an enterohepatic recirculation of approximately 4%. Enhancement during the distribution phase of the contrast agent mainly depends on the vascularity, while enhancement on delayed images 20 minutes after administration is characterized by the selective uptake of the contrast agent by the hepatocytes. Non-hepatic tissue (e.g. liver metastases) shows no contrast enhancement on delayed images [15,17,21,23-26] (Figure 1a + c). The signal intensity of liver parenchyma on the delayed images correlates with the functioning state of the according hepatocytes with a decreased signal intensity in dysfunctional liver parenchyma [27]. Thus a MRI based volumetric assessment of liver parenchyma damage is possible.

### Image registration

Quantitative analysis of hepatocellular dysfunction and edema in areas exposed to focal high dose rate brachytherapy was performed using T1-w GRE 20 minutes post intravenous administration of Gd-EOB-DTPA and T2-w UTSE with fat suppression, respectively. Follow-up imaging was performed at four follow-up time-points (3 days, 6, 12 and 24 weeks after brachytherapy) leading to a total number of 92 MRI scans in 23 patients.

For image fusion of the follow-up MRI scans with the according treatment plan (based on the planning CT data set), MRI data was transferred to the treatment planning system. BrachyVision^® ^offers an isoscalar local semi-automated point based 3D-3D image registration. Match points were defined on corresponding landmarks such as branches of the portal vein to enable fusion of MR and planning CT/dosimetry data. Landmarks were restricted to liver structures.

**Figure 2:**
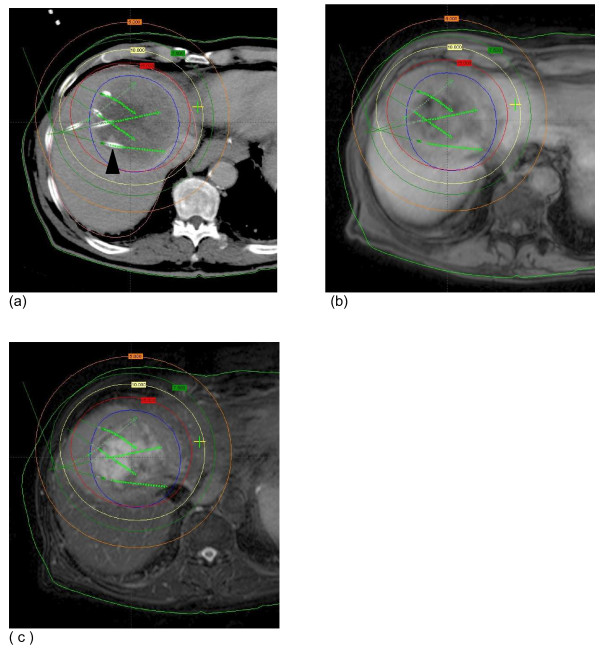
**Image fusion of planning CT/dosimetry and follow-up MRI**. (a) Contrast-enhanced CT after CT-guided positioning of 5 brachytherapy catheters in a colorectal metastasis (one catheter is labeled with a black arrowhead, the other catheter positions in cranial or caudal planes are indicated by green arrows). Isodoses lines the CTV (blue circle) after dosimetry with BrachyVision^® ^(b + c). MRI 3 months after brachytherapy: T1-w gradient echo 20 minutes post i.v. application of Gd-EOB DTPA (b) and T2-w UTSE FS (c) showing image fusion with the treatment planning CT.

A linear interpolation was performed automatically by BrachyVision^® ^to match the varying slice thicknesses of T2-w MRI (8 mm) and CT (5 mm). As a result of this procedure, BrachyVision^® ^simultaneously displayed the treatment plan as well as the anatomical structures of the MRI scan (Figure 2a -c). The absolute registration error was always less than 5 mm. A retrospective registration of pre-treatment MRI with planning CT/dosimetry to obtain the CTV as basline in MRI was deemed inappropriate due to a possible additional registration error. As already stated the tumor extent in pre treatment MRI was respected in dosimetry planning.

### Quantitative analysis

**Figure 3:**
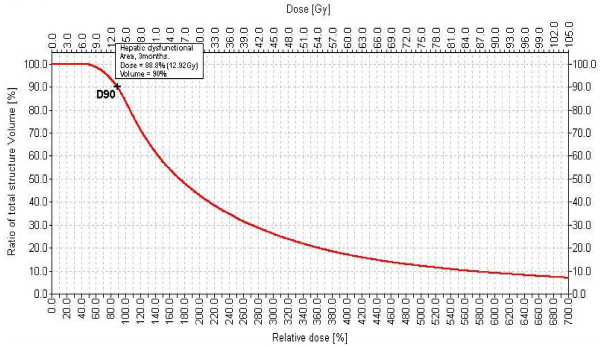
**Dose-Volume-Histogram of nonfunctioning liver volume**. Dose volume histogram of nonfunctioning liver volume in a patient 3 months after HDR brachytherapy. (D90: the dose applied to at least 90% of the volume, in this case 12.92 Gy.)

On all of the T1-w late Gd-EOB-DTPA as well as the T2-w enhanced images an experienced GI Radiologist digitally outlined the border of hypointensity on T1-w images (loss of hepatocyte function as displayed by diminished Gd-EOB-DTPA uptake) or hyperintensity on T2-w images (edema) around the irradiated liver tumor (referred to as "pseudolesion (including the irradiated tumor)" in the following). Pre-existing peritumoral changes could be excluded on the pre treatment MRI. Based on the total 3D data set of these volumes, the Brachyvision^® ^software calculated a dose-volume histogram. As a result, we determined the percentage of each pseudolesion receiving a specific dose. We specified the threshold dose for either edema or function loss as the D_90_, i.e. the dose achieved in at least 90% of the pseudolesion volume (Figure 3).

As an additional descriptor, we determined the volume of each pseudolesion in relation to the former intrahepatic 10Gy isodose volume. This additional volumetric approach was performed as an independent verification of the previously described methodology.

### Laboratory analysis

One day prior to the intervention and during follow up we assessed the following laboratory parameters: bilirubin, aspartate aminotransferase, alanine aminotransferase, alkaline phosphatase, albumin, international normalized ratio, and cholinesterase. Laboratory parameters were graded according to the 'Common Terminology Criteria for Adverse Events' (CTCAE version 4.02, National Cancer Institute, USA).

No patient presented evidence of liver function degradation prior to therapy.

### Factors of influence

Following factors were recorded and tested for influence on the minimal dose provoking edema or hepatocyte function loss (i.e. the dose at 6 weeks): patient age, the liver volume, the clinical target volume, the liver volume which was exposed with more than 5Gy or 10Gy, the source factor, the irradiation time, the number of catheters applied, history of chemotherapy.

### Statistical analysis

Results of continuous data are displayed as medians and ranges and/or lower and upper quartile, results of frequency data as counts and percentages.

For two-group comparisons of the medians two-sided Wilcoxon rank sum tests were used. Correlations were evaluated using a two sided Pearson correlation test.

A p < 0.05 was considered to be statistically significant.

For statistical analysis the software 'Statistical Package for the Social Sciences' (IBM SPSS, Version 17.0, Somers, NY, USA) was used.

## Results

The registration error of the landmarks after image fusion of the planning CT to the follow-up MRI was as follows: in median 0.95 mm (0.53-1.5), 1.82 mm (0.22-2.91), 1.79 mm (0.81-2.91) and 2.04 mm (0.85-5) for the T1 weighted sequences at 3 days, 6, 12 and 24 weeks, respectively. For the T2 weighted images, the registration error was in median 0.98 mm (0.59-1.3), 1.75 mm (0.79-3.65), 1.89 mm (0.81-3.89) and 1.98 mm (0.98-3.53), respectively.

**Figure 4:**
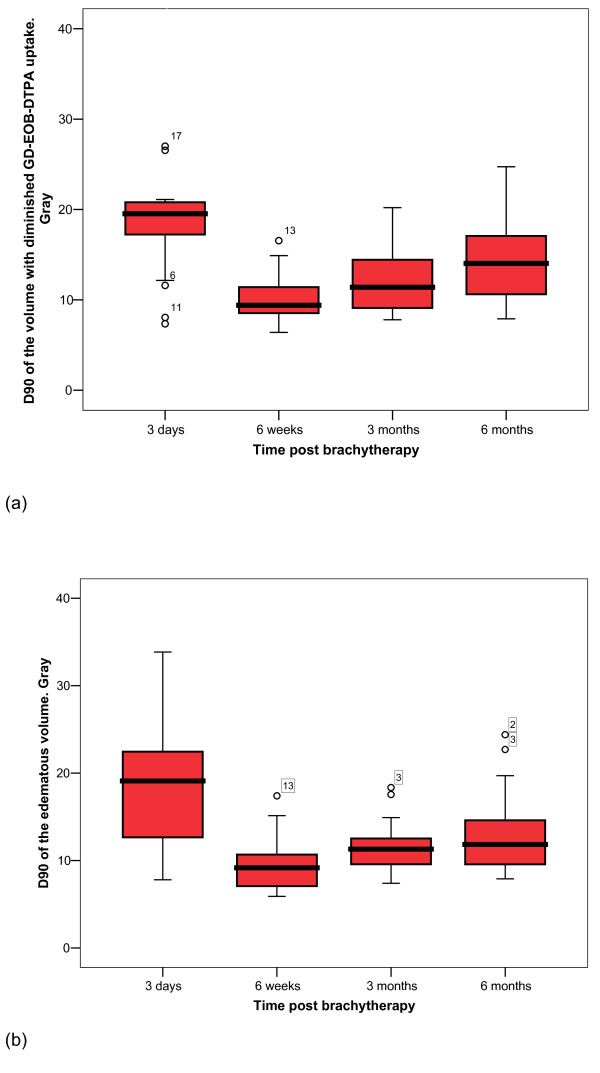
**Boxplot of threshold doses of hepatocyte function loss and edema over time**. (a) Hepatocyte function loss over time relative to dose exposition. (b) Development of the according edema.

Figure 4a + b display the development of hepatocyte dysfunction or hepatic edema in correlation to the isodose distribution. Three days after brachytherapy, the D_90 _for hepatocyte function loss reached a median of 19.5Gy (7.4-53.1, Q_25_: 16.9, Q_75_: 21.1), i.e. doses ≥19.5Gy provoked a state of hepatocyte dysfunction. At six weeks, the isosurface had increased significantly to a median of 9.4 Gy (6.4-16.6, Q_25_: 8.5, Q_75_: 11.7; p < 0.001), i.e. protracted toxic effects lowered the threshold dose for hepatic dysfunction to 9.4Gy. Between 6 and 12 weeks, the dysfunctional volume had decreased significantly to a median of 11.4Gy (7.8-20.2, Q_25_: 9, Q_75_: 14.5; p = 0.002). After 24 weeks, the isosurface decreased further to a median of 14 Gy (7.9-24.7, Q_25_: 10.5, Q_75_: 17.6; p = 0.002).

Between three days and 6 weeks, the extension of the edema increased significantly from the 19.1Gy (7.8-33.8, Q_25_: 12.3, Q_75_: 22.7) isosurface to 9.2Gy at median (5.9-17.4, Q_25_: 6.8, Q_75_: 10.9; p < 0.001). After this peak a significant decrease occurred between 6 and 12 weeks (median 11.3Gy, 7.4-18.4, Q_25_: 9.3, Q_75_: 13.1; p = 0.002). After 24 weeks, the edematous tissue isosurface shrank to a median of 11.8 Gy (7.9-24.3, Q_25_: 9.3, Q_75_: 14.8; p = 0.018).

At three days post brachytherapy, the minimal dose leading to edema tended to be less compared to the dose provoking focal hepatocyte function loss (p = 0.055). No differences between the doses provoking focal hepatic dysfunction or edema were noted at 6, 12 and 24 weeks (p = 0.158, 0.212 and 0.128, respectively).

**Figure 5:**
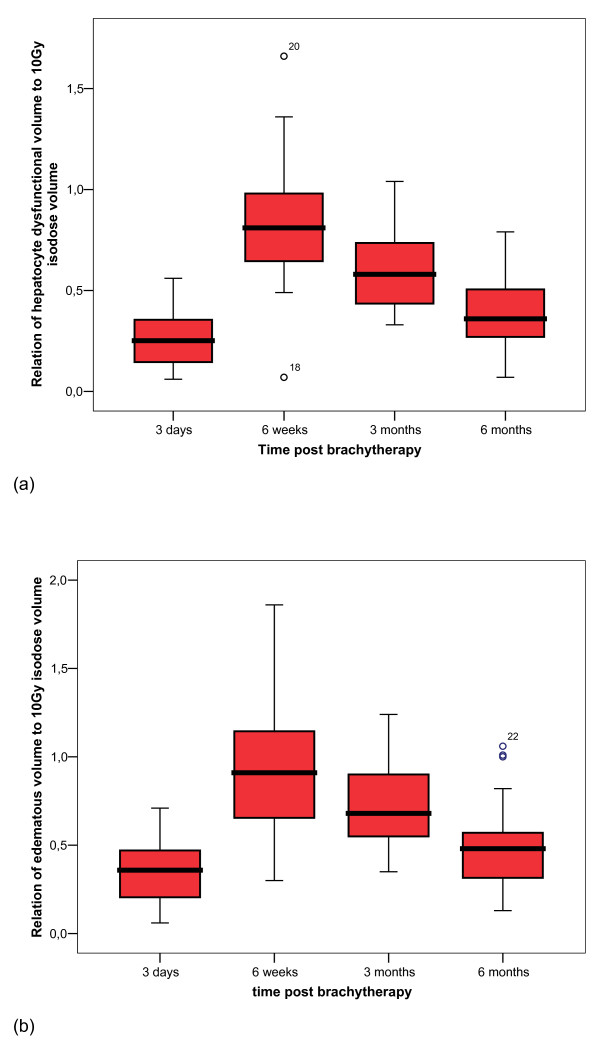
**Boxplot of volume of hepatocyte function loss and edema over time**. Development of hepatic function loss (a) and edema (b) around the irradiated tumor relative to the 10 Gy isodose volume (liver parenchyma only).

Figure 5a + b illustrate the development of the volume of focal hepatocyte function loss or edema post brachytherapy in correlation to the liver volume which was exposed with more than 10Gy. The relative volume of hepatocyte function loss increased significantly between three days and 6 weeks (p < 0.001) with an extend to 81% of the 10Gy volume in median after 6 weeks (Q_25_: 61%, Q_75_: 100%). After 12 weeks, the decline of the volume with hepatocyte function loss was significant compared to 6 weeks (58%, Q_25_: 43%, Q_75_: 76%; p < 0.001), and again after 24 weeks when compared to 12 weeks (36%, Q_25_: 27%, Q_75_: 51%; p < 0.001). Between three days and 6 weeks, edema volume increased significantly (6 weeks: 91%, Q_25_: 63, Q_75_: 116%; p < 0.001). From week 6 to week 12, edema volume shrank significantly (68%, Q_25_: 54%, Q_75_: 91%; p = 0.001) and continued to week 24 with a significant decrease (48%, Q_25_: 31%, Q_75_: 60%; p < 0.001). At all time points the size of the edema volume surmounted the size of the volume of hepatocyte dysfunction in relation to the 10 Gy volume (p < 0.05).

We found no statistical correlation between the minimal dose provoking edema or hepatocyte function loss (i.e. the dose at 6 weeks) and: patient age, the liver volume, the clinical target volume, the liver volume which was exposed with more than 5Gy or 10Gy, the source factor, the irradiation time, the number of catheters applied, or a history of chemotherapy.

No patient included in this study demonstrated a severe impairment of liver function as measured by the above named laboratory parameters before CT guided brachytherapy. No severe acute or late toxicity were observed after CT guided brachytherapy. Liver function tests graded according to the 'common terminology criteria for adverse events' (CTCAE) version 4.02 never exceeded grade 2 toxicities during follow up.

## Discussion

Quantitative data on the radiation threshold dose of hepatic tissue is scarce compared to other late-responding tissues. In this study we used regional uptake of the hepatocyte specific MRI contrast media Gd-EOB-DTPA as a surrogate parameter for hepatic function after HDR irradiation of small liver volumes. Thus we were able to determine the hepatocyte tolerance dose in vivo. Small volumes of hepatic parenchyma irradiated with more than 9.4Gy revealed a non functioning state after six weeks. After six months, recovery of hepatic parenchyma led to a threshold dose of 14 Gy for hepatic dysfunction.

**Figure 6:**
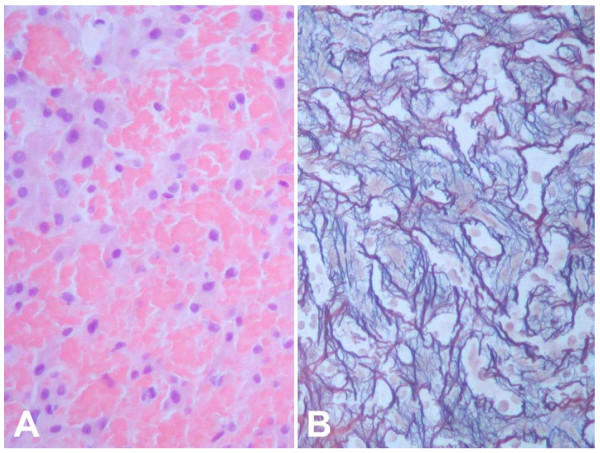
**Histological specimen: liver tissue after radiation exposure**. Liver biopsy in an area exposed to approximately 20 Gy two months earlier. Severe sinusoidal congestion with atrophy of hepatocytes (A) and increased perisinusoidal reticulin deposition (B). Hematoxylin-eosin (A) and Gomori's silver stain (B), original magnification: x200. Biopsy was taken to rule out local recurrence.

The histological appearance of radiation induced liver disease indicates that endothelial injury and subsequent obstruction of centrilobular venules and sinusoids are the key events in the pathogenesis of radiation injury of the liver. Larger veins are frequently spared [7,12,13,28]. The pathological lesion resembles veno-occlusive disease, frequently seen after total body irradiation in induction therapy prior to bone marrow transplantation [29-31]. This initial injury is followed by a wide, edematous subendothelial zone, resembling deposits of fibrin-related aggregates and fragmented red cells in the subendothelial zone. Early deposition of fibrinogen is frequently found without platelet accumulation. These aggregates, as well as the intramural entrapment of fluid and cellular debris, progressively occlude the hepatic venous outflow by intraluminal sinusoidal fibrous material, subendothelial collagen fibers and foamy cells [7,13,28] (Figure 6). Experimental studies on hepatic radiation injury support the theory that the endothelial lining of venules and sinusoids is far more sensitive to radiation than hepatocytes [32-34].

However, changes of uptake of a hepatocyte specific contrast media illuminate the final path of the radiation injury, i.e. these changes visualize areas of a dysfunctional hepatic system, not necessarily individual hepatocyte dysfunction [27]. The model does not differentiate whether parenchymal (hepatocytes) or non-parenchymal cells (endothelial-, Kupffer- and Ito-cells) play the key role in the development of focal hepatic dysfunction. Hence, our model must be interpreted as the reaction of a liver functioning unit to irradiation.

Therapeutic irradiation of liver malignancies is restricted by hepatic tolerance with the clinical endpoint liver function loss. In total or large volume irradiation of the liver, major complications such as RILD have frequently been described [6,7,10,11]. However, the parallel functional structure of the liver may cover a loss of extensive liver volume without clinical symptoms such as after hepatectomy. A comparison between data of total or large volume liver irradiation using the clinical endpoint RILD or liver failure has to be differentiated from small volume irradiation as executed in our study. CT-guided brachytherapy as well as stereotactic irradiation of liver malignancies are locally circumscribed radiotherapies designed to spare healthy liver parenchyma. In a clinical setting, a relatively small rim of liver parenchyma around the CTV is exposed to high irradiation doses. This limited loss of functioning hepatic tissue is generally tolerated well without any impairment of the whole organ function as seen by unchanged liver function parameters during follow-up [35]. However, caution should be exercised if the total volume of unexposed liver is small such as

• in patients scheduled for synchronous multifocal irradiation of liver malignancies because of overlapping isodoses of the single lesions.

• in patients scheduled for irradiation of a large tumor volume in small livers.

• in patients scheduled for irradiation with a history of partial liver resection because of a potentially small residual parenchymal volume.

• In patients with chronic and/or degenerative liver diseases (such as cirrhosis).

An increase of the incidence of clinical RILD has been observed in total liver irradiation when doses 30-55Gy are delivered applying conventional fractionation schemes [6,7,10-14,28]. These fractionated tolerance doses translate into single-fraction doses of 11.25 up to 14Gy, respectively, according to the linear-quadratic model and an assumed α/β of 3Gy for liver tissue [11]. These threshold values correspond well to our own data on focal liver dysfunction after single fraction small volume irradiation and correspond well to observed clinical significant hepatic injury after doses as low as 10Gy in patients undergoing a single fractioned total body or abdominal irradiation and chemotherapy prior to bone marrow transplantation [29,30,36]. However, an unequal dose rate of the different approaches has to be considered.

The reaction of small liver volumes to irradiation has also been studied by other groups. Herfarth et al. examined hepatic tolerance after applying stereotactic single-fraction irradiation to liver malignancies with data derived from follow-up contrast-enhanced CT. The authors observed a focal reaction at a dose minimum of mean 13.7Gy (range 8.9 -19.2Gy) [5]. In principle, contrast enhanced CT will most likely demonstrate the venous outflow occlusion after radiation exposure. This approach visualizes the underlying pathophysiological change provoking focal liver function degradation. Consequently, the results of this workgroup match well with the data derived from our model applying hepatocyte directed contrast agent in MRI, visualizing the effect of venous outflow occlusion with distraction of a liver function unit as described above. It remains questionable whether the higher standard deviation of their results may be explained by a smaller sensitivity of the CT model as compared to MRI [5,8].

In the present study we not only sought to add further data on hepatic dose tolerance, but to improve the study concept applied previously that had used the first generation hepatocyte-directed contrast agent Gd-BOPTA [8]. While the pharmacodynamics of Gd-BOPTA are similar to those of Gd-EOB-DTPA, the pharmacokinetics differ significantly. The biliary excretion rate of Gd-BOPTA is just 0.6 to 4% compared to 41.6 to 51.2% in Gd-EOB-DTPA [15-18]. This results in a higher signal-to-noise-ratio in Gd-EOB-DTPA and consequently to a better demarcation of liver lesions and non-functional parenchyma. The results presented in this study determining the threshold dose of hepatocytes support the data from the precursor study using Gd-BOPTA [8]. Apart from the different hepatocyte specific contrast agent used (Gd-BOPTA vs. Gd EOB DTPA) in MRI follow up, the design of these two studies was similar. The minimal tolerance dose to high dose irradiation in the precursor study was determined with 9.5Gy in median (6-14.5, Q_25_: 8, Q_75_: 12) after six weeks (n = 25), compared to 9.4Gy in median (6.4-16.6, Q_25_: 8.5, Q_75_: 11.7) in the present study. Both studies showed a strong recuperation of dysfunctional liver parenchyma after six months with a residual non-functioning liver parenchyma in areas irradiated with formerly more than 15.2Gy in median (7.5-23, Q_25_: 12, Q_75_: 18) and 14 Gy in median (7.9-24.7, Q_25_: 10.5, Q_75_: 17.6) at present [8].

However, we did not reach our goal to decrease the relatively high variation from the precursor study employing Gd-BOPTA, despite the superior imaging properties of Gd-EOB-DTPA. We attribute this failure to the fact that individual differences in hepatic dose tolerance do not preferably reflect flaws of the imaging model, but rather unknown factors of individual predisposition. In other words, data derived from different models (including CT or MR imaging) consistently shows deviations of individual hepatic dose tolerance to small volume single fraction irradiation.

## Conclusion

In summary, our study supports previous data on hepatic tolerance doses after single fraction, high dose rate small volume irradiation. We conclude that the threshold dose to induce a focal loss of liver function is about 10 Gy after 6 weeks. We confirmed previously observed strong recuperation with a threshold dose of 14 Gy after 6 months. These results should be considered specifically in cases where more than one liver lesion shall be irradiated, or in patients with a history of focal liver irradiation. Further investigation is warranted to assess the correlation between deminished uptake of Gd-EOB-DTPA in the affected liver volume and clinical symptoms as liver function degradation before routine use of Gd-EOB-DTPA can be recommended in this matter.

## List of abbreviations

RILD: Radiation Induced Liver Disease; CT: Computed Tomography; 3D: Three Dimensional; MRI: Magnetic Resonance Imaging; Gd-BOPTA: Gadobenate dimeglumine; HDR: High Dose Rate; Gd-EOB-DTPA: Gadolinium ethoxybenzyl diethylenetriaminepentaacetate; CTV: Clinical Target Volume; T2-w: T2-weighted; UTSE: Ultrafast Spinecho; TE: Time to Echo; TR: Time to Repetition; T1-w: T1-weighted; GRE: Gradient Echo.

## Conflict of interest

JR receives grants for consulting and research from Bayer Healthcare. No stock holder or shares. All other authors: there is no actual or potential conflicts of interest, sources of financial support, corporate involvement, patent holdings, etc. for each author to report.

## Authors' contributions

MS and RS participated in the study design and drafted the manuscript; KM performed image fusion and screened patients for inclusion; CW participated in the design of the study; TK performed histological staining and corrected the manuscript; SL performed image fusion and screened patients for inclusion; MP served as reader; PW participated in study design; JR conceived of the study and paticipated in its design and coordination. All authors read and approved the final manuscript.
